# Lactase-Treated A2 Milk as a Feasible Conventional Milk Alternative: Results of a Randomized Controlled Crossover Trial to Assess Tolerance, Gastrointestinal Distress, and Preference for Milks Varying in Casein Types and Lactose Content

**DOI:** 10.3390/nu17121946

**Published:** 2025-06-06

**Authors:** Laura A. Robinson, Aidan M. Cavanah, Sarah Lennon, Madison L. Mattingly, Derick A. Anglin, Melissa D. Boersma, Michael D. Roberts, Andrew Dandridge Frugé

**Affiliations:** 1 Department of Nutritional Sciences, Auburn University, Auburn, AL 36849, USA; 2 College of Nursing, Auburn University, Auburn, AL 36849, USA; 3 School of Kinesiology, Auburn University, Auburn, AL 36849, USA; 4 Department of Chemistry and Biochemistry, Auburn University, Auburn, AL 36849, USA

**Keywords:** fluid milk, lactose, breath hydrogen, gastrointestinal tolerance, A2 milk, beta-casein

## Abstract

**Background:** Previous research indicates that gastrointestinal discomfort from milk consumption may be attributable to A1 β-casein, rather than lactose intolerance alone. A2 milk (free of A1 β-casein) consumption may result in fewer symptoms compared to conventional milk containing both A1/A2 β-casein. **Objective:** In this five-week, double-blind, double-crossover study, we assessed the physiological responses to doses escalating in volume of lactose-free conventional milk (Lactaid), A2 milk, and lactose-free A2 milk in fluid milk-avoiding participants. **Methods:** Each milk type was consumed over three separate weeks with three increasing doses across five days per week, >one week washout. Gastrointestinal symptoms, blood glucose, and breath gases were monitored for twenty-four, two-, and three-hours post-consumption, respectively. Sensory evaluation was completed for each sample. **Results:** Fifty-three participants consented and were randomized, with forty-eight participants completing the study. Overall, symptoms were minimal. On Days 1 and 3, lower ratings of bloating and flatulence were observed in A2 compared to lactose-free A2. Breath hydrogen responses reflected lactose content, but were higher in lactose-free A2 than Lactaid on Day 5. Thirty-three participants were deemed lactose-intolerant and had higher fasting and average breath hydrogen for all samples. The only symptom corresponding to the increase in breath hydrogen among these participants was flatulence after A2 consumption. Surprisingly, flatulence was apparently higher for lactose-tolerant individuals when consuming Lactaid compared to A2. **Conclusions:** These findings suggest that adults who avoid conventional fluid milk consumption may experience minimal GI discomfort from lactose-free and/or A1-free milks.

## 1. Introduction

Gastrointestinal (GI) symptoms such as bloating, diarrhea, and gas following the consumption of fluid milk are commonly attributed to lactose malabsorption, a condition affecting approximately 68% of the global population [1,2]. Lactose malabsorption results from insufficient production of lactase, the enzyme required to hydrolyze lactose in the small intestine [2]. This enzymatic deficiency leads to the fermentation of unabsorbed lactose by intestinal microbiota, resulting in GI discomfort [1,3]. For individuals with lactose malabsorption, lactose-free milk provides a nutritionally similar alternative while minimizing GI discomfort [4,5]. However, emerging evidence suggests that lactose intolerance may not be the sole cause of GI symptoms associated with fluid milk consumption [6]. A1 and A2 β-casein, the two predominant protein variants in cow’s milk, may also play a significant role. The distinction between A1 and A2 β-casein arises from a single amino acid difference at position 67 of the β-casein polypeptide chain, where histidine in A1 is replaced by proline in A2. This substitution affects the protein’s structure, and during digestion, A1 β-casein releases β-casomorphin-7 (BCM-7), an opioid peptide that has been implicated in gastrointestinal and systemic effects, including increased GI symptoms in sensitive individuals. In contrast, A2 β-casein does not produce BCM-7, and milk containing only A2 β-casein has been associated with reduced GI symptoms in sensitive individuals [6,7,8,9]. Epidemiological studies have suggested associations between A1 β-casein consumption and increased risks of certain chronic diseases, including type 1 diabetes and cardiovascular conditions, although more research is needed to establish causality [10].

Randomized controlled trials have demonstrated that individuals consuming A2 milk experience fewer GI symptoms, such as nausea, abdominal pain, and fecal urgency, compared to those consuming conventional milk, which contains both A1 and A2 β-casein [11,12]. Furthermore, studies report reduced breath hydrogen levels, a marker of lactose malabsorption, and improved stool consistency, stool frequency, and inflammatory markers following A2 milk consumption [13]. Notably, cognitive improvements have been observed in sensitive populations consuming A2 milk, suggesting broader systemic benefits [14]. Additionally, the A1 β-casein variant has been associated with increased gastrointestinal inflammation and delayed intestinal transit, effects not observed with A2 β-casein consumption [9]. Given this evidence, it is hypothesized that milk-induced GI symptoms may stem not only from lactose intolerance but also from the presence of A1 β-casein. Substituting conventional milk with A2 β-casein milk may offer therapeutic benefits for individuals who experience GI discomfort.

Despite these findings, the current body of research on the differential effects of A1 and A2 β-casein is limited. Only a few human studies have directly compared the gastrointestinal impacts of these protein variants, and results have been inconsistent due to variations in study design, populations, and outcome measures. Moreover, many studies have not adequately controlled for lactose content, making it challenging to isolate the effects of β-casein variants from those of lactose. This gap in the literature underscores the need for well-designed studies that specifically examine the gastrointestinal responses to A1 and A2 β-casein, independent of lactose content.

This study aimed to evaluate the effects of milk with varying proportions of A1 and A2 β-casein and lactose on GI distress and tolerance in adults who regularly avoid fluid milk. We conducted a five-week trial to assess GI and physiological responses to escalating doses of three types of milk: lactose-free conventional milk, A2 milk, and lactose-free A2 milk. The primary outcome was change in GI distress over a 24 h period following milk consumption. Secondary outcomes included breath hydrogen, blood glucose, and milk preference ratings. We hypothesized that lactose-free A2 milk would result in the least GI symptoms and maldigestion, given the absence of both lactose and A1 β-casein.

## 2. Materials and Methods

### 2.1. Study Design and Interventions

The primary aim of this study was to evaluate the impact of milk containing different types of casein (A1 and A2) and lactose on GI distress and tolerance in individuals who avoid consuming fluid milk. The main outcome measure was differences in subjective GI distress. Secondary objectives included changes in breath hydrogen, breath methane, and blood glucose following the consumption of each milk type and dose.

**Figure 1:**
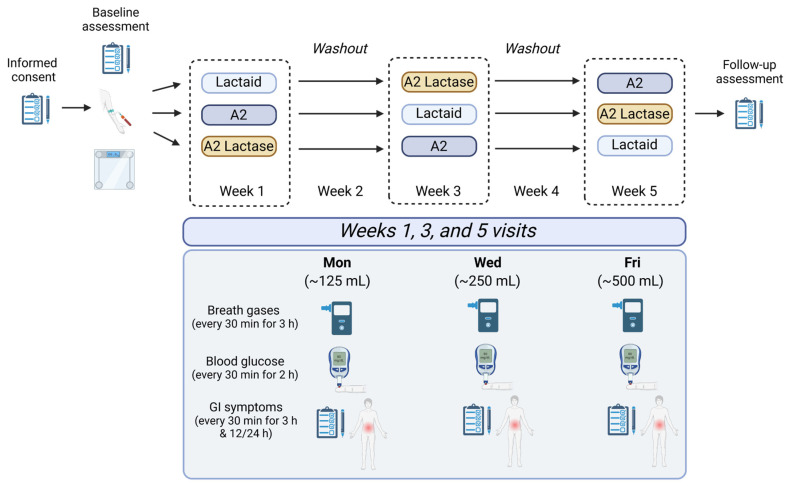
Schematic of study design and flow. Participants (*n* = 48) consumed nine total samples over a five-week period. Conventional milk pre-treated with lactase (Lactaid), milk from a2/a2 cattle (A2), and lactase-treated A2 (A2 Lactase) were provided blinded in ascending doses each week. Gastrointestinal symptoms, blood glucose, and breath gases were assessed before and after consumption of each sample.

The study was pre-registered at ClinicalTrials.gov (identifier: NCT06315517) and its protocol was approved by the Auburn University IRB (IRB #: 23-552 MR 2404). The trial was conducted in accordance with the Declaration of Helsinki. This study employed a randomized, controlled, double-blind, double-crossover trial design, with all testing conducted at the Auburn University School of Kinesiology. Figure 1 provides an overview of the study design, and the assessments conducted at each visit. After completing informed consent and baseline visits, participants were randomized to one of six study sequences. Baseline measurements included an 8 h fasted intravenous blood draw, assessment of height and weight using a Tanita BC-568 InnerScan Segmental Body Composition Monitor (Tanita Corp USA, Arlington Heights, IL, USA), medical history, and medication documentation.

Participants reported to the laboratory during three separate weeks, three sessions per week, each lasting two hours, with a total study duration of five weeks. Participants were asked to come to each test day fasted for at least two hours. During each test day, 24 h dietary recalls were collected, and sensory evaluations of milk samples were completed. Breath samples were collected to measure hydrogen and methane levels prior to sample consumption, then every 30 min for three hours. Capillary finger sticks were conducted to measure blood glucose levels every 30 min for two hours. GI symptoms were assessed using Visual Analog Scales over the following 24 h. A washout period of seven to ten days was implemented between test phases to minimize carryover effects.

At the end of the study, participants attended a follow-up assessment where they were informed about the specific weeks they received each milk sample, and an exit interview was conducted to determine their overall sample preference.

#### 2.1.1. Participants

Participants were recruited using Institutional Review Board (IRB)-approved flyers which directed individuals to a Qualtrics-hosted eligibility screener. Eligible participants were males or females aged 18 years or older who self-reported avoiding fluid milk consumption and were able to read and speak English. Exclusion criteria included a diagnosis of milk protein allergy, Crohn’s disease, ulcerative colitis, celiac disease, peptic ulcers, or gastroparesis; use of antibiotic or antiviral medication, chemotherapy treatment, and/or the use of investigational drugs within 30 days prior to the study; and being pregnant or lactating during the time of the study. Recruitment was conducted from early May 2024 to late July 2024.

Upon providing consent, participants were assigned an identification number. After completing the baseline visit, they were randomized into intervention sequences using sealedenvelope.com, stratified by body mass index (BMI; <34 or >34 kg/m^2^) and race (non-Hispanic white or another race).

#### 2.1.2. Sample Preparation

Milk samples utilized in this study included ultra-high temperature (UHT)-pasteurized 2% lactose-free cow’s milk, containing 60% A1 and 40% A2 β-casein (Lactaid^®^; McNeil Nutritionals, LLC, Fort Washington, PA, USA), and 2% A2 cow’s milk (The A2 Milk Company, Boulder, CO, USA), both obtained from a local Kroger grocery store. A2 Lactase samples were treated as follows: 59 fluid ounce cartons of A2 milk were treated with 20 drops of lactase (Milkaid lactase enzyme drops; Crosscare Limited, Dublin, Ireland). The treated milk was stored for 48 h to ensure adequate enzymatic hydrolysis of lactose [15]. For administration to participants, milk samples were prepared in clear plastic cups sealed with plastic wrap and labeled with participant identification numbers at the beginning of each test day and stored at 4 degrees Celsius. Each sample provided to participants was based on their baseline body weight: 50 mg of casein per kilogram of body weight on Day 1 (~125 mL of milk), 100 mg of casein per kilogram on Day 3 (~250 mL of milk), and 200 mg of casein per kilogram on Day 5 (~500 mL of milk). This resulted in varying volumes per participant: 3–9 fluid ounces on Day 1, 6–18 fluid ounces on Day 3, and 12–35 fluid ounces on Day 5.

### 2.2. Study Measures

#### 2.2.1. Gastrointestinal Symptom Scores

Gastrointestinal (GI) symptoms were assessed at baseline, immediately before the milk intervention, and 30, 60, 90, 120, 150, and 180 min and 12 and 24 h after consumption. The symptoms were evaluated using a standard 100 mm VAS, with the far left, labeled 0, indicating not at all and the far right, labeled 10, indicating severe symptoms. Individual VASs were completed for abdominal pain, bloating, flatulence, and diarrhea, with the option to write in and rate additional symptoms.

#### 2.2.2. Breath Gases

Breath samples were collected from participants in duplicate immediately before sample consumption, and 30, 60, 90, 120, 150, and 180 min post-consumption. Samples were obtained using a patented closed-system breath collection device, which captured exhaled air into a glass test tube. The collected samples were subsequently analyzed using a QuinTron breath analyzer (QuinTron Instrument Company, Inc., New Berlin, WI, USA). Hydrogen, methane, carbon dioxide, and the corresponding correction factors were recorded for each sample [13].

#### 2.2.3. Blood Glucose Levels

Blood glucose levels were measured immediately before milk consumption and 30, 60, 90, and 120 min after consumption [16] using Medline EvenCare ProView Blood Glucose Meters and Glucose Test Strips (Medline Industries, Inc., Mundelein, IL, USA). At each time point, two measurements were taken and averaged to obtain a final value. A third sample was tested if measurements differed more than 5 mg/dL.

#### 2.2.4. Milk Sensory Scale

Participants completed a 5-Point JAR (Just-About-Right) Cow Milk Sensory Scale to evaluate the hedonic attributes of the milk samples consumed. They were instructed to mark an ‘x’ next to the option that best described their experience of each attribute, including color, milkiness, creaminess, mouthfeel, aftertaste, and overall rating. JAR scales are bipolar attribute scales, with a midpoint labeled ‘just-about-right (JAR)’ and endpoints representing two opposite descriptors, such as ‘too strong’ and ‘too weak’ [17,18].

#### 2.2.5. Lactose Tolerance

Lactose intolerance was determined based on changes in breath hydrogen (H_2_) levels over the 180 min period following the ingestion each of the lactose-containing A2 milk samples. Participants were classified as lactose-intolerant if they exhibited an increase of ≥20 parts per million (ppm) in H_2_ from baseline levels at any time point during the post-consumption period [3].

#### 2.2.6. Milk Sample Composition

Composition of milk samples measured via Nanoflow liquid chromatography and UPLC with ESI-MS/MS are seen in Table 1. Milk sugars were assessed using the methods of Chavez-Servin et al. [19] and Yang et al. [20]. Exactly 250 µL of milk was added to a 50 mL centrifuge tube and 5 mL of ethanol was added. The mixture was sonicated and heated to 60 °C for 20 min. Next, 500 µL of a solution consisting of 0.36 g of K_4_[Fe(CN)_6_]·3H_2_O in 10 mL water (Carrez reagent 1) was added and vortexed for 1 min. Then, we added 500 µL of Carrez reagent 2, namely 0.72 g ZnSO_4_·7H_2_O in 10 mL water, and vortexed for 1 min. Further, 5 mL of acetonitrile was added and vortexed to mix. Finally, 13.75 mL of 50% ethanol in water was added and mixed. The suspension was centrifuged at 4619 rpm for 10 min at 4 °C. The solutions were decanted and frozen until analysis, where 50 µL was diluted into 950 µL acetonitrile.

Analysis was performed on a Vanquish UHPLC system (Thermo Fisher, Waltham, MA, USA) coupled with a quadrupole orbitrap mass spectrometer (Orbitrap Exploris 120, Thermo) with electrospray ionization (H-ESI) in negative mode using Xcalibur software (V4.4.16.14). Injection of 10 µL of the sample was made on an Amide column (ACQUITY UPLC^®^ BEH Amide, 3.5 µm, 2.1 × 50 mm, Waters, Milford, MA, USA) held at 60 °C with a 600 μL/min flow rate of mobile phase solution A (99.9% water with 0.1% ammonia) and solution B (99.9% acetonitrile water with 0.1% ammonia). Glucose and galactose had similar retention times and were therefore not differentiated. The gradient began at 95% B, was held for 1 min, followed by a linear ramp to 40% B at 11 min, held for 1 min, then back to 97% B for a total analysis time of 23 min. The flow was diverted to waste from start to 0.6 min and after 8 min. The MS scan range was 100–1000 *m*/*z* with a resolution of 120,000, standard AGC target, 70% RF lens, maximum injection time auto, and EASY-IC run-start on. The spray voltage was 2100 V, the ion transfer tube temperature was 2800 °C, and the vaporizer temperature was 250 °C. Data was processed using Xcalibur.

To determine milk proteins, milk samples were defatted by skimming the top fatty layer off the sample after centrifugation at 2600× *g* for 20 min [21]. Protein was concentrated by centrifugation at 16,100× *g* for 20 min and the supernatant was discarded. Protein was solubilized in 50 mM ammonium bicarbonate (ABC) and a Bradford assay was performed. A total of 25 µg of protein was transferred to a low-protein binding tube (Eppendorf cat# 022431081), diluted with 40 µL ABC, and reduced with 1 µL of 250 mM DTT via incubation at 56 °C for 20 min. The capping reagent, 1.35 µL of 550 mM iodoacetamide, was added to the sample and incubated in dark conditions for 20 min at room temperature. Thermolysin was added at a ratio of 1:25 to the protein sample and incubated at 60 °C for 4 h [22]. The samples were cleaned up with Stage-Tips created with Empore C18 material by washing with methanol, 0.1% TFA, adding sample, washing with 0.1% TFA, and eluting with 3 × 10 µL 80% acetonitrile and 20% 0.1% TFA. Speedvacuum centrifugation was performed at medium heat for 3 h to remove acetonitrile, then diluted to a total of 20 µL with 0.1% TFA [23].

Samples were analyzed on an Orbitrap Explorus 240 mass spectrometer (Thermo Fisher, Waltham, MA, USA) equipped with an EASY-Spray PepMap RSLC C18 2 µm, 100 Å column, and a nano UltiMate 3000 binary pump and autosampler with an Acclaim PepMap 100 Å C18 3 µm trapping column. The gradient solvent system consisted of 0.1% (*v*/*v*) FA in water (buffer A) and 0.1% (*v*/*v*) FA in 80% acetonitrile 20% water (buffer B). Peptide samples were separated at a flow rate of 300 nL/min with a 60 min segmented gradient as follows: 3.5% (*v*/*v*) buffer B for 5 min, to 36% buffer B at 40 min, to 60% buffer B at 45 min, to 98% buffer B at 48 min and held for 6 min, to 3.5% B in one min and 5 min re-equilibration at 3.5% B. Full MS scans were acquired with an *m*/*z* range of 300−1500 and mass resolution of 60,000 with advanced peak width determination enabled and RunStartEasyIC. The normalized automatic gain control (AGC) target was set to 300%, and the RF lens was set to 70%, with scans up to 20 ms. MS/MS acquisition was performed in top 10 mode. The charge state filter was set to 2–5, the application mode was set to peptide, the intensity filter was set to a minimum of 5000, and automatic dynamic exclusion was utilized. The resolved fragments were scanned at a mass resolution of 30,000 and a normalized AGC target value of 75% and normalized HCD collision energy of 30% were used. The isolation window was 1.6 *m*/*z*. Data was searched against *Bos taurus* and *Bos indicus* fasta files using Proteome Discoverer 2.5 [22].

### 2.3. Adverse Events

Adverse events occurring at any time during the study up to the final assessment were documented. Participants were instructed to report any adverse events on the GI symptom questionnaire or to notify the research staff by telephone or during study visits.

### 2.4. Statistical Analyses

Statistical analyses were performed using SPSS v29 (IBM Corp, Armonk, NY, USA) and graphs were plotted using GraphPad Prism (Version 10.1; San Diego, CA, USA). To account for non-normality of dependent variables, a mixed generalized linear model was used for continuous variable analyses, incorporating milk type, time, and testing day as fixed factors, with Sidak adjustments applied to control for multiple comparisons. Statistical significance was set at α ≤ 0.05.

## 3. Results

**Figure 2:**
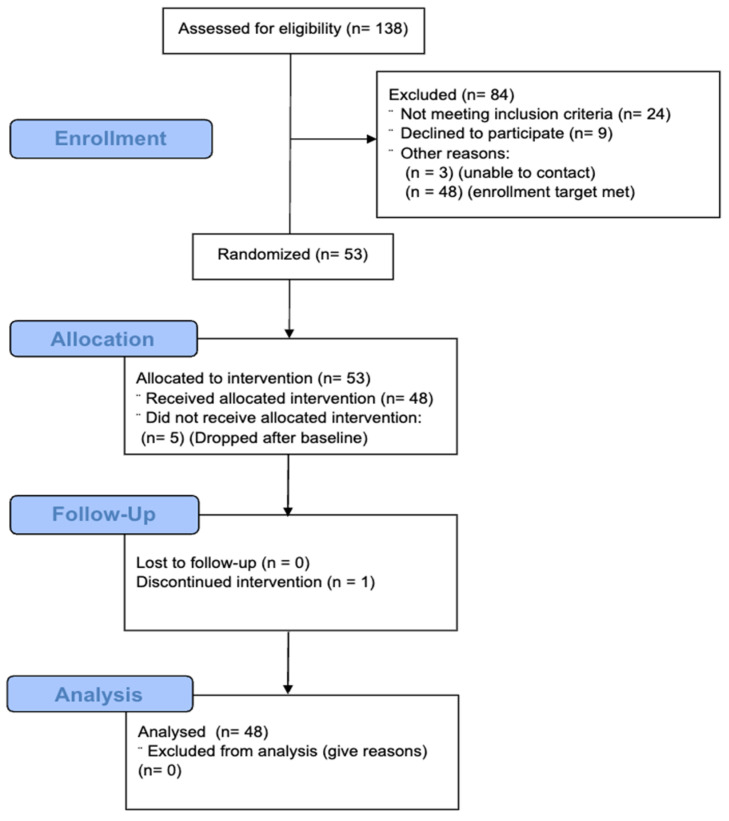
Consolidated Standards of Reporting Trials (CONSORT) diagram of study flow.

A total of 138 individuals were screened for eligibility, with 84 excluded due to not meeting inclusion criteria (*n* = 24), declining participation (*n* = 9), or inability to contact (*n* = 3), finally reaching the enrollment target (*n* = 48) (Figure 2).

### 3.1. Participant Eligibility, Enrollment, and Randomization

Participants were randomly assigned to sequences of milk consumption order by body mass index (BMI) (<34 or >34 kg/m^2^), sex (Male or Female) and race (White or Other). Of the 53 randomized participants, 48 received the allocated intervention, with 5 withdrawing consent prior to beginning the intervention and 1 participant discontinuing after starting the intervention.

The baseline characteristics of the 48 participants are shown in Table 2. The sample included 16 males and 32 females (*p* = 0.089), with a mean age of 31 ± 9 years and mean BMI of 27.4 ± 5.6 kg/m^2^. Participants were white (*n* = 22), Asian (*n* = 9), Black (*n* = 8), Hispanic (*n* = 8), Latino (*n* = 1), and White/Latino (*n* = 1), with no significant differences in ethnicity (*p* = 0.195). No significant differences were observed in age (*p* = 0.814) or BMI (*p* = 0.405) across randomization sequences. Thirty-three (68.8%) participants were deemed lactose-intolerant, which were unequally distributed across randomization sequences (*p* < 0.001).

### 3.2. Consumer Preference Across Samples

In our sample of non-milk-drinkers, average ratings for all attributes were predominantly rated as ‘just right’, with overall preferences falling between ‘neutral’ and ‘like’. Few differences were observed between the samples across individual attributes. However, significant variations were noted in milkiness and aftertaste between lactose-free samples (A2 lactase and Lactaid) and regular A2 milk.

**Figure 3:**
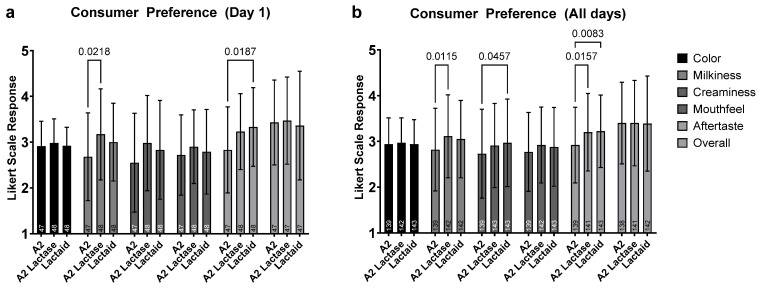
Consumer preferences measured by the 5-point Just-About-Right (JAR) sensory scale for (**a**) Day 1 of each sample, and (**b**) Days 1, 3, and 5 combined. For panel (**a**), all 48 participants rated all three samples on Day 1 (*n* = 141). For panel (**b**), all 48 participants rated all three serving sizes (Days 1, 3, 5) of all three samples (*n* = 423).

On Day 1 (Figure 3a), A2 lactase was rated significantly higher in milkiness compared to A2 milk (*p* = 0.0218), while Lactaid exhibited a significantly stronger aftertaste (*p* = 0.0187). When data from all testing days were aggregated (Figure 3b), A2 lactase maintained a significantly higher milkiness rating than A2 milk (*p* = 0.0115). Lactaid was marginally, but significantly, creamier than A2 milk (*p* = 0.0457). Additionally, both Lactaid and A2 lactase received stronger aftertaste ratings compared to A2 milk (*p* = 0.0083 and *p* = 0.0157, respectively).

### 3.3. Gastrointestinal Symptoms in Response to Milk Types

**Figure 4:**
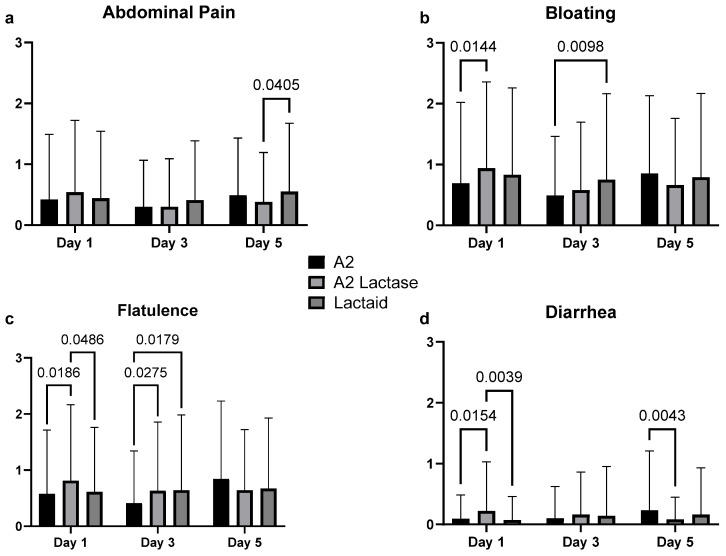
Aggregated gastrointestinal symptoms reported by study participants consuming fluid milk samples varying in casein type and lactose content across days, measured by Visual Analog Scale (VAS): (**a**) abdominal pain, (**b**) bloating, (**c**) flatulence, and (**d**) diarrhea. Each day includes symptoms reported prior to consuming the sample and post-consumption at +30, +60, +90, +120, and +180 min, and +12 and +24 h (*n* = 3667 for each panel).

Self-reported gastrointestinal symptoms, including abdominal pain, bloating, flatulence, and diarrhea, were averaged across all milk types for each test day, as shown in Figure 4. Generalized linear models revealed significant variations in the severity of these symptoms across different test days. However, there was no consistent pattern indicating that any specific milk type consistently resulted in higher gastrointestinal symptom scores. Mean symptom scores for all participants across all categories remained below 1 out of 10, suggesting no clinically significant differences in gastrointestinal responses among the milk types.

A2 lactase had marginally lower abdominal pain scores on Day 5 (*p* = 0.0405), and significantly lower diarrhea on Day 5 (*p* = 0.0043). On the contrary, A2 lactase resulted in significantly higher diarrhea scores on Day 1 compared to both A2 and Lactaid (*p* = 0.0154 and *p* = 0.0039). A2 milk had significantly lower bloating scores compared to A2 lactase on Day 1 (*p* = 0.0144) and Lactaid on Day 3 (*p* = 0.0098). A2 milk also had significantly lower flatulence scores on Day 1 compared to A2 lactase (*p* = 0.0186) and Day 3 compared to both A2 lactase and Lactaid (*p* = 0.0275 and *p* = 0.0179).

### 3.4. Breath Hydrogen in Response to Milk Type over Time

**Figure 5:**
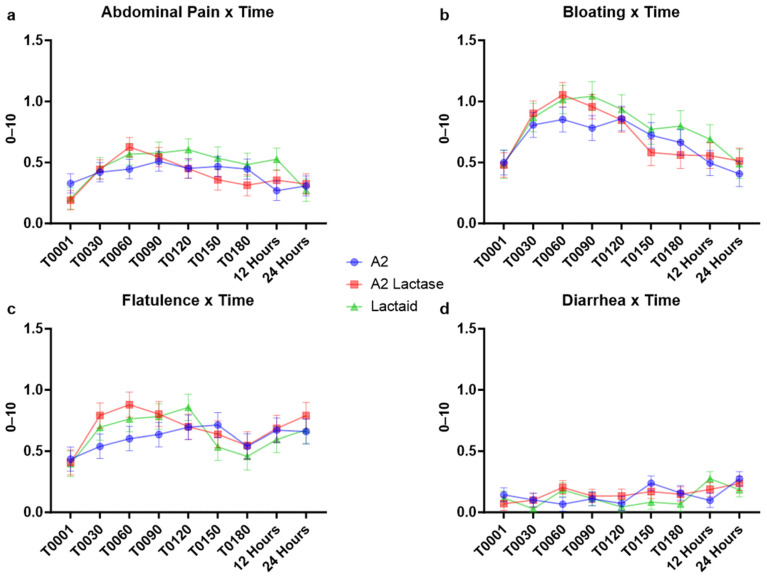
Aggregated gastrointestinal symptoms across a 24 h time period, pre- and post-consumption of fluid milk samples varying in casein type and lactose content, measured by 1-10 Visual Analog Scale (VAS): (**a**) abdominal pain by time, (**b**) bloating by time, (**c**) flatulence by time, and (**d**) diarrhea by time (*n* = 3667 for each panel).

Neither abdominal pain, bloating, flatulence, nor diarrhea showed statistical difference in severity across a 24 h period, as shown in Figure 5.

**Figure 6:**
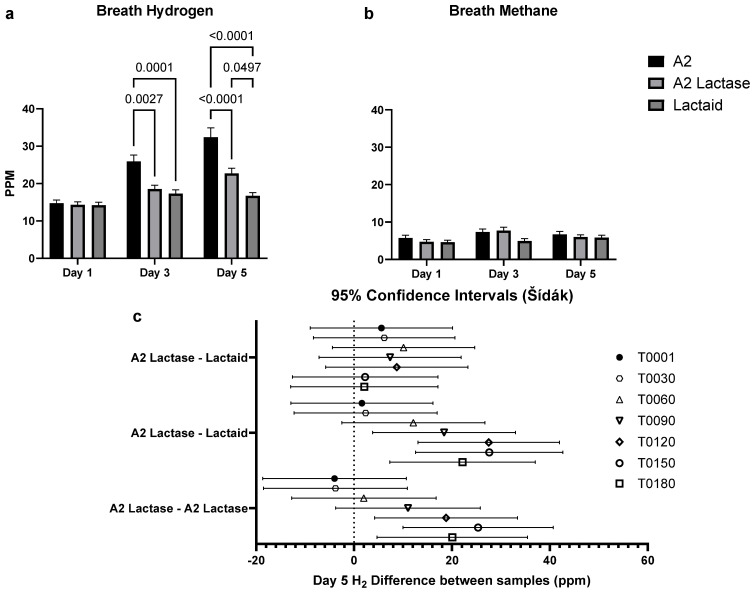
Comparison of H_2_ and CH_4_ production between A2, A2 Lactase, and Lactaid milk: (**a**) total H_2_ levels measured on Days 1, 3, and 5, expressed in parts per million (PPM); (**b**) total CH_4_ levels measured on Days 1, 3, and 5, expressed in parts per million (PPM); and (**c**) total H_2_ difference on Day 5, between samples across time points (*n* = 2931).

On Days 3 and 5, A2 milk consumption resulted in significantly higher H_2_ levels compared to the other samples (Figure 6a). Post hoc analysis using Sidak’s test revealed no significant differences between A2 lactase and Lactaid on Day 5 (Figure 6c). However, on Day 3 (Figure 6a), a significant difference was observed between A2 Lactase and Lactaid (*p* = 0.0497). Overall, A2 milk demonstrated statistically significant differences on both Day 3 and Day 5, as illustrated in Figure 6a,c, particularly when comparing A2 with Lactaid and A2 Lactase. There was no significant difference in breath methane levels across all test days (Figure 6b).

### 3.5. Blood Glucose Response to Milk Samples

Blood glucose levels were elevated at 30 min and returned to near baseline for all samples at all time points (Figure S1). On Day 5, the lactase-treated samples had higher non-significant excursions at 30 min but did not have greater rebound effects at one hour post-consumption.

### 3.6. Breath Hydrogen and Flatulence Response to Milk Type Between Lactose Intolerant Individuals

**Figure 7:**
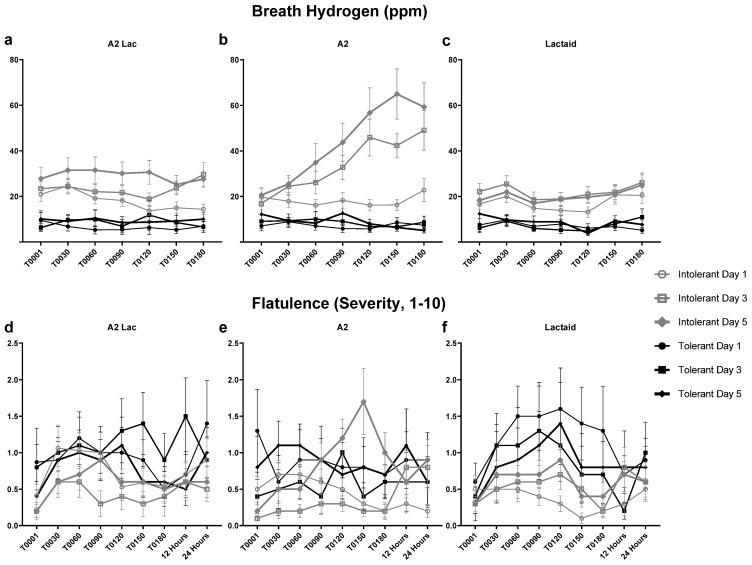
Comparison of hydrogen production: (**a**) Post A2 Lactase consumption H_2_ levels measured on across time, (**b**) Post A2 consumption H_2_ levels measured on across time, (**c**) Post Lactaid consumption H_2_ levels measured on across time, measured in PPM and flatulence severity, (**d**) Post A2 Lactase consumption over time (**e**) Post A2 consumption over time, (**f**) Post Lactaid consumption over time, measured on a scale of 1–10 VAS, between likely lactose-intolerant (*n* = 33) and lactose-tolerant (*n* = 15) participants.

Lactose-intolerant participants exhibited significantly higher breath H_2_ levels after consuming A2 milk on days 3 and 5 compared to tolerant participants, as illustrated in Figure 7a–c. Despite their elevated baseline breath H_2_ levels, no significant differences in post-consumption H_2_ levels were observed between lactose-intolerant and lactose-tolerant participants for the A2 lactase or Lactaid samples. For the lactose-containing sample, breath H_2_ levels increased significantly on Days 3 and 5, likely due to the larger doses administered on these days. In contrast, H_2_ levels remained consistently low for lactose-tolerant participants and all lactose-free samples, indicating minimal physiological response. Among gastrointestinal symptoms, flatulence was the only symptom that correlated with breath H_2_ levels; however, no significant associations were identified, as seen in Figure 7d–f. Although lactose-tolerant participants reported greater flatulence severity after consuming Lactaid, no significant differences in flatulence severity were observed between lactose-intolerant and lactose-tolerant participants over time. However, lactose-tolerant participants reported higher flatulence levels for lactose-free samples, a pattern not observed among lactose-intolerant individuals.

## 4. Discussion

This study aimed to compare the effects of lactose-free conventional milk (30% A1/70% A2 β-casein), A2 milk (100% A2 β-casein), and lactose-free A2 milk on gastrointestinal distress, physiological responses, and milk preference in adults who self-reported avoiding fluid milk. Contrary to our hypothesis, A2 lactase milk did not consistently result in lower GI symptoms compared to the other milk samples. While A2 lactase caused fewer abdominal and diarrhea symptoms on the Day 5 testing days, this effect was not consistently observed across other symptoms or test days. A2 milk appeared to cause fewer GI symptoms overall on Days 1 and 3; however, symptom severity increased by Day 5, likely due to the greater lactose content in the sample. Additionally, no significant differences were observed in the fluctuation of GI symptom scores over the 24 h post-consumption data collection periods.

Consumer preference did not differ significantly between the samples, although the lactose-free samples were rated higher in milkiness, creaminess, and aftertaste compared to A2 milk. Breath hydrogen levels were significantly higher after A2 milk consumption on Days 3 and 5 compared to both A2 lactase and Lactaid milk, indicating lactose malabsorption. Unexpectedly, H_2_ levels also increased significantly on Day 5 for A2 lactase milk compared to Lactaid, raising questions about potential malabsorption of another component in the A2 lactase sample. On Day 5, breath H_2_ levels rose significantly around 90 min post-consumption for A2 lactase compared to Lactaid and around 120 min post-consumption for A2 lactase compared to A2 milk. Flatulence was the only GI symptom that correlated with breath H_2_ levels in lactose-intolerant individuals. Notably, lactose-intolerant participants began with higher baseline breath H_2_ levels than lactose-tolerant participants. However, lactose-tolerant individuals reported higher flatulence levels for both lactose-free samples, a pattern not observed among lactose-intolerant participants. These findings suggest that GI symptoms were not solely dependent on lactose malabsorption, as indicated by the breath hydrogen levels.

Previous randomized controlled trials have demonstrated that lactose-intolerant individuals experience reduced GI symptoms when consuming A2 milk compared to conventional milk containing both A1 and A2 β-casein. For example, one study investigating females with self-reported lactose intolerance reported reduced symptoms of nausea and fecal urgency following A2 milk consumption compared to conventional milk, but not lactose-free conventional milk. Interestingly, the same study found that non-lactose dairy-intolerant females experienced rapid-onset GI symptoms regardless of milk type, without a corresponding increase in breath H_2_ levels, suggesting mechanisms independent of lactose malabsorption [11]. In a crossover pilot study comparing A1 versus A2 β-casein, Ho S et al. found that non-lactose-intolerant participants consuming A1 β-casein milk exhibited significantly higher Bristol Stool Scale consistency values compared with A2 β-casein. There was also a significant positive association between abdominal pain and stool consistency on the A1 diet, but not the A2 diet [24]. Furthermore, some individuals may be susceptible to A1 β-casein, as evidenced by higher fecal calprotectin values and associated intolerance measures. Another randomized crossover study, A2 milk increased bloating and loose stools compared to A1/A2 milk in individuals who experienced GI discomfort after milk consumption. A2 milk caused less abdominal pain, fecal urgency, and borborygmus compared to A1/A2 milk in digestive symptom questionnaires. In addition, fecal calprotectin also increased less after consumption of A2 milk compared to A1/A2 milk, and this change was more pronounced in males than in females [25]. Ramakrishnan et al. also compared GI symptoms post A2 milk consumption to conventional milk, Jersey milk, and Lactaid. Significant reductions in abdominal pain scores among lactose-intolerant participants following A2 milk consumption compared to conventional milk, though no differences were observed with Jersey milk (containing 25%/75% A1/A2 β-casein) and Lactaid. Other GI symptoms showed no significant differences six hours post-consumption. Breath hydrogen levels were significantly lower after A2 milk consumption compared to conventional milk [13]. Reduced GI symptoms have also been observed in Chinese children consuming A2 milk, including decreased stool frequency, and improved stool consistency [12]. Similarly, Jiang et al. reported significantly greater digestive discomfort and prolonged gastrointestinal transit times [14].

## 5. Limitations and Future Research

This study has several limitations. We excluded habitual milk drinkers, but not individuals who consumed other forms of dairy. Thus, we did not instruct participants to avoid dairy products during the intervention period. This could have influenced baseline breath hydrogen levels and GI symptom data, potentially confounding the results. Additionally, variations in participants’ overall dietary habits during the study period may have affected GI symptoms and physiological markers, as other food components or meal patterns could interact with milk consumption and breath gas production. We also did not perform a lactose challenge test to definitively confirm lactose intolerance among participants. As a result, our method of determining lactose intolerance may not accurately represent the true lactose-intolerant individuals within the participant pool, potentially impacting the generalizability of the findings. Furthermore, psychological factors, such as participants’ pre-existing beliefs or expectations about lactose intolerance or the perceived effects of specific milk types, may have influenced their symptom reporting, introducing potential placebo effects. Lastly, the sample included 16 males and 32 females, indicating a gender imbalance. This disproportion may limit the generalizability of the findings across sexes, as physiological and hormonal differences can influence gastrointestinal responses to milk consumption. These limitations highlight the need for further research to validate and expand upon our findings under more controlled and comprehensive conditions. Finally, we did not take whole blood samples throughout the study and were unable to measure BCM-7, a peptide derived from A1 β-casein that has been associated with reduced GI symptoms in sensitive individuals [6,7,8]. This exclusion limits our ability to directly assess its potential role in the observed outcomes.

## 6. Conclusions

This study provides novel insights as the first to directly compare the effects of A2 milk, A2 lactase milk, and Lactaid milk on gastrointestinal symptoms, physiological responses, and consumer preferences in adults who avoid fluid milk. While A2 and lactose-free A2 milk demonstrated potential for reducing GI symptoms compared to Lactaid milk, these effects were not universally consistent. Interestingly, breath hydrogen levels and GI symptoms suggest that other factors beyond lactose malabsorption may contribute to milk intolerance, particularly for lactose-free products. Given the nutrient density of bovine milk and its potential contributions to bone health, metabolic regulation, and chronic disease prevention, future research should focus on elucidating the mechanisms by which A1 and A2 β-casein influence digestive health [26,27]. Additionally, it would be beneficial for future studies to compare the differences in GI symptoms and physiological responses between both conventional and A2 milk, lactose-free versions, such as A2 lactase and Lactaid milk, to better understand their respective impacts and potential benefits for sensitive individuals. Nonetheless, our results suggest that the samples tested were well accepted and tolerated by adults who do not habitually drink fluid milk.

## Figures and Tables

**Table 1:** Composition of milk samples measured via Nanoflow liquid chromatography and UPLC with ESI-MS/MS.

Nutrients	A2	A2 Lactase	Lactaid
Lactose, g/240 mL	12.9	0.0	0.3
Glucose and galactose, g/240 mL	0.0	15.0	13.4
A1 β-casein as % total β-casein	0.0	0.0	23.9

**Table 2:** Baseline characteristics of participants (N = 48) randomized to consume milk samples varying in casein type and lactose content. Three sample types consumed in random order yielded six total sequences denoted as A to F below.

Sequence	A	B	C	D	E	F	All	*p*
	N = 9	N = 8	N = 9	N = 8	N = 7	N = 7	N = 48	
Sex								0.089
Male	4 (44.4%)	1 (12.5%)	3 (33.3%)	1 (12.5%)	5 (71.4%)	1 (14.3%)	15 (31.3%)	
Female	5 (55.6%)	7 (87.5%)	6 (66.7%)	7 (87.5%)	2 (28.6%)	6(85.7%)	33 (68.8%)	
Ethnicity								0.195
White	4 (44.4%)	4 (50.0%)	3 (33.3%)	4 (50.0%)	3 (42.9%)	4 (57.1%)	22 (45.8%)	
Black	0 (0.0%)	4 (50.0%)	1 (11.1%)	1 (12.5%)	1 (14.3%)	0 (0.0%)	7 (14.6%)	
Asian	2 (22.2%)	0 (0.0%)	3 (33.3%)	1 (12.5%)	3 (42.9%)	0 (0.0%)	9 (18.8%)	
Hispanic	2 (22.2%)	0 (0.0%)	1 (11.1%)	2 (25.0%)	0 (0.0%)	3 (42.9%)	8 (16.7%)	
Latino	0 (0.0%)	0 (0.0%)	1 (11.1%)	0 (0.0%)	0 (0.0%)	0 (0.0%)	1 (2.1%)	
White/Latino	1 (11.1%)	0 (0.0%)	0 (0.0%)	0 (0.0%)	0 (0.0%)	0 (0.0%)	1 (2.1%)	
Age	31 (7.14)	33 (9.55)	29 (5.36)	31 (8.21)	25 (3.10)	33 (16.40)	31 (8.97)	0.814
BMI	29.0 (7.45)	25.0 (6.73)	26.2 (3.34)	28.1 (6.46)	27.1 (3.42)	28.1 (5.84)	27.2 (5.70)	0.405
Lactose-Intolerant	7 (77.8%)	6 (75%)	6 (66.7%)	3 (37.5%)	5 (71.4%)	6 (85.7%)	33 (68.8%)	<0.001

## Data Availability

Data is available from the corresponding author upon reasonable request.

## Supplementary Materials

The following supporting information can be downloaded at: https://www.mdpi.com/article/10.3390/nu17121946/s1. Supplementary Figure S1. Comparison of blood glucose levels between A2, A2 Lactase, and Lactaid milk.

## Abbreviations

The following abbreviations are used in this manuscript:

ABC	Ammonium bicarbonate
AGC	Automatic gain control
A2 Lac	Lactose-free A2 milk
ANOVA	Analysis of variance
BCM-7	β-casomorphin-7
BMI	Body mass index
DTT	Dithiothreitol
ESI-MS/MS	Electrospray ionization tandem mass spectrometry
FA	Formic acid
GI	Gastrointestinal
H_2_	Hydrogen
HCD	Higher-energy collisional dissociation
IRB	Institutional Review Board
JAR	Just-About-Right
Lac	Lactaid (lactose-free conventional milk)
m/z	Mass-to-charge ratio
MDPI	Multidisciplinary Digital Publishing Institute
MS	Mass spectrometry
PPM	Parts per million
RF	Radio frequency
SPSS	Statistical Package for the Social Sciences
SD	Standard deviation
TFA	Trifluoroacetic acid
UHPLC	Ultra-high-performance liquid chromatography
UHT	Ultra-high temperature
VAS	Visual analog scale
