# Supplementary material for: Lactase-Treated A2 Milk as a Feasible Conventional Milk Alternative: Results of a Randomized Controlled Crossover Trial to Assess Tolerance, Gastrointestinal Distress, and Preference for Milks Varying in Casein Types and Lactose Content
Source: Nutrients. 2025 Jun 6;17(12):1946. doi: 10.3390/nu17121946 (PMC12196342; doi:10.3390/nu17121946)
Supplement: Supplementary file 1 [file nutrients-17-01946-s001.zip › nutrients-3662562-supplementary.pdf]

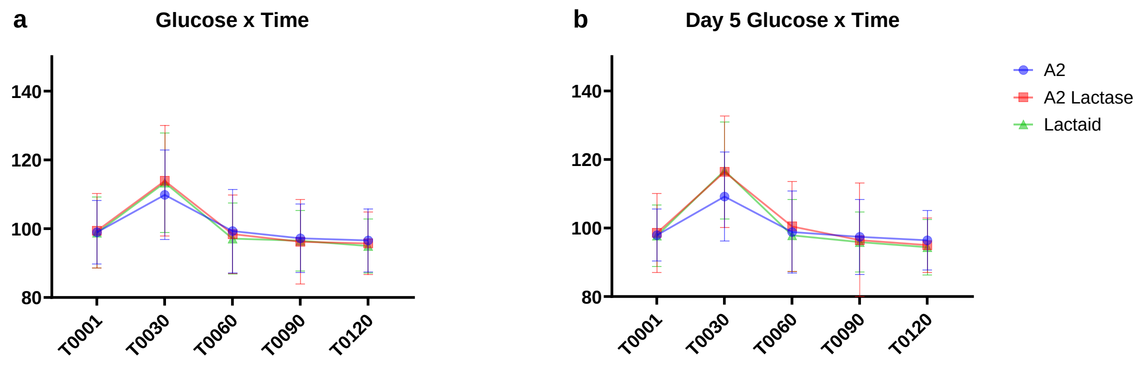

Supplementary Figure S1. Comparison of blood glucose levels between A2, A2 Lactase, and Lactaid milk: (**a**) Aggregate total blood glucose levels across time, and (**b**) Day 5 total blood glucose levels over time post-milk consumption.
